# Electrostatic Charge on Flying Hummingbirds and Its Potential Role in Pollination

**DOI:** 10.1371/journal.pone.0138003

**Published:** 2015-09-30

**Authors:** Marc Badger, Victor Manuel Ortega-Jimenez, Lisa von Rabenau, Ashley Smiley, Robert Dudley

**Affiliations:** 1 Department of Integrative Biology, University of California, Berkeley, California, United States of America; 2 Department of Mechanical Engineering, University of California, Berkeley, California, United States of America; 3 Department of Biology, University of New Mexico, Albuquerque, New Mexico, United States of America; 4 Smithsonian Tropical Research Institute, Balboa, Republic of Panama; University of Nebraska-Lincoln, UNITED STATES

## Abstract

Electrostatic phenomena are known to enhance both wind- and insect-mediated pollination, but have not yet been described for nectar-feeding vertebrates. Here we demonstrate that wild Anna's Hummingbirds (*Calypte anna*) can carry positive charges up to 800 pC while in flight (mean ± s.d.: 66 ± 129 pC). Triboelectric charging obtained by rubbing an isolated hummingbird wing against various plant structures generated charges up to 700 pC. A metal hummingbird model charged to 400 pC induced bending of floral stamens in four plants (*Nicotiana*, *Hemerocallis*, *Penstemon*, and *Aloe* spp.), and also attracted falling *Lycopodium* spores at distances of < 2 mm. Electrostatic forces may therefore influence pollen transfer onto nectar-feeding birds.

## Introduction

Electrostatic forces can play an important role in pollination mediated by both wind and animal vectors [1]. Because of a gradient in electrical potential between pollen and floral structures [2], charged pollen carried by the wind can settle more effectively on the stigma than can uncharged pollen. Similarly, electrical charge carried by insects may be sufficient to increase the number of pollen grains transferred to their bodies during floral visits [3]. For example, placing a charged metal model or an actual tethered insect close to a grounded source of pollen grain can induce attraction [4], and presumably increase the likelihood of subsequent pollen transfer by an insect to another flower. These electrostatic means of pollination are currently being explored for agricultural applications (see [5]).

By contrast, pollination by bats or birds has not yet been examined relative to the potential role of electrostatics, although 86% of extant angiosperm families have species pollinated by vertebrates [6], and some dedicated nectarivores such as hummingbirds can potentially visit thousands of flowers per day [7]. There are currently no data available on the magnitude of the electrical charge typically carried by a flying vertebrate, and it is unknown whether such charge would be sufficient to enhance pollination. Here, we present experimental evidence demonstrating that free-flying Anna’s Hummingbirds (*Calypte anna*) can accumulate electrical charge sufficient to attract pollen grains, and also to induce displacement of floral filaments and anthers towards the beak and head of the birds. We also demonstrate that wing frictional contact with petals and with plant leaves can generate high charges that potentially facilitate pollen transfer.

## Materials and Methods

### Hummingbird charge

We measured the electrical charge carried by wild hovering Anna’s Hummingbirds under different humidities and air temperatures from 8–24 November 2013. Charge was measured using a combination feeder/sensor consisting of a plastic syringe filled with sugar water (~20% sucrose by volume) tipped with a 1.5 cm segment of copper tube (4 mm in diameter). An aluminum disc (3 cm in diameter) covered with orange and red electrical tape was attached to the copper tube to simulate an artificial flower. The feeder was suspended within a square Faraday cage (35 cm × 35 cm × 35 cm) using a metal clamp; the cage was grounded and open on one side. The measurement and ground cables of a charge sensor (Code CRG-BTA, Vernier Software & Technology) were connected to the copper feeder tip and to the Faraday cage, respectively. Charge data were acquired using a USB sensor interface (Go!Link, Vernier Software & Technology) and associated Logger Lite Software sampling at a frequency of 10 Hz. The Faraday cage and charge sensor were mounted in a upper-floor window of the Valley Life Sciences Building, University of California, Berkeley, such that the open side of the cage was open to the outdoors and attracted wild hummingbirds to the feeder. An observer (VMO-J) had a clear sight of each bird visiting the feeder, and nearly all of the visits were performed by males. As individual hummingbirds were not marked during the experiment, the total number of birds sampled was unknown and multiple visits by the same individual were possible.

Relative humidity (±1%) and air temperature (±0.1°C) within the cube during each hummingbird visit were recorded using a combination thermo-hygrometer (Taylor USA, model 1523). For one day (22 Nov. 2013), relative humidity was lower than the sensor’s minimal measurement point (20%), and humidity for that day was instead obtained from a nearby Berkeley weather station (KCABERKE31). Using this experimental feeder, we measured the charge of wild hummingbirds as they hover-fed under different conditions of humidity and temperature. Baseline drift of the sensor was removed from the sample trace using a linear model, and net charge was taken to be calculated as the difference in sensor output prior to and following a hummingbird visit. Pooling all data points, we then modeled charge as a second degree polynomial function of humidity and temperature, including day of measurement as a random factor.

### Stamen attraction

**Fig 1:**
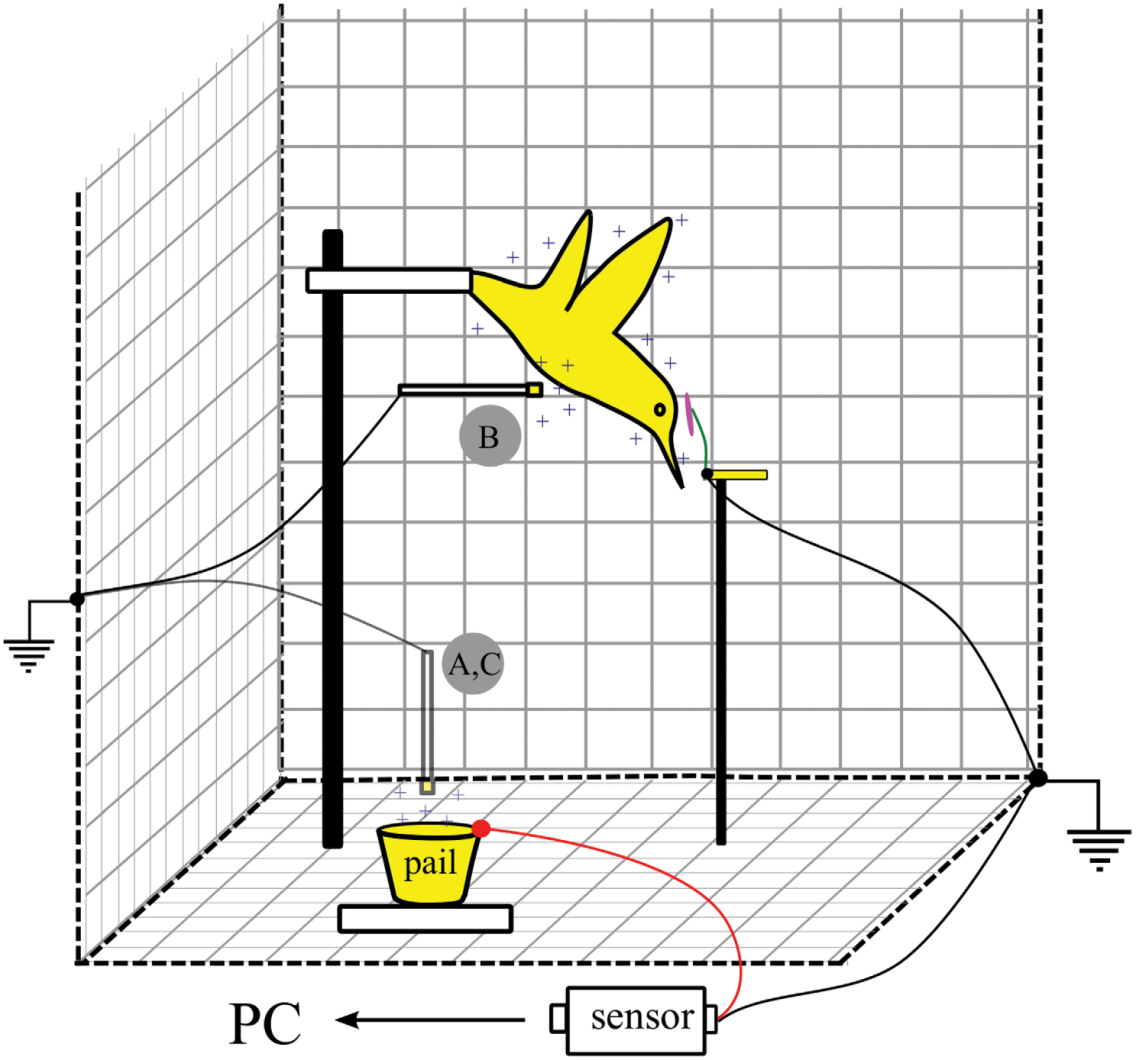
Experimental setup used to study electrostatic attraction of floral stamens and *Lycopodium* spores to a charged metal hummingbird model. Metal objects are indicated in yellow; floral anthers and filaments are depicted in pink and green, respectively. The hummingbird model is oriented vertically so as to avoid gravitational displacement of spores towards the model. (A) The metal tip of the rachis was first charged by contact with a portable Van de Graaff generator and introduced into a copper pail. (B) Charge was then transferred to the hummingbird model. (C) The metal tip of the rachis was then immediately reintroduced into the copper pail. See text for details.

Attraction forces exerted on floral structures by charged hummingbirds were studied using stamens freshly detached from flowers. We fastened a metal hummingbird model to a support stand using a plastic rod as an insulator, and placed it within the aforementioned Faraday cage (Fig 1). We then mounted a single grounded and detached floral stamen by its base using a brass tube (0.12 × 2 cm), and positioned it near to the model's forehead. Both hummingbird and stamen were rotated 90° relative to their natural orientation so that gravitational forces did not contribute to filament bending (see Fig 1).

We then transferred a known electrical charge to the model using an insulated feather rachis with an attached metal tip. The other end of the rachis was fastened to a metal cap and was grounded with a metal wire. The hummingbird model was discharged before each trial. The metal tip of the rachis was then charged by contact with a portable Van de Graaff generator (Unitech Toys, Foster City, CA), was immediately introduced into a copper pail (2 cm height × 2 cm diameter) without making contact with the pail walls, and then was subsequently touched to the hummingbird model for several seconds. We then reintroduced the metal tip of the rachis into the copper pail to transfer any remaining charge. The copper pail was connected to the aforementioned charge sensor, which allowed us to measure net charge on the metal tip of the rachis. Total charge on the hummingbird model was calculated as the difference between the first and second charge measurements from the copper pail (Fig 1).

We transferred charges ranging from 250–800 pC to the hummingbird model, and then slowly displaced the stamen towards the model's forehead until physical contact between the two was visible. All trials were filmed at 60 frames/s (X-PRI, AOS Technologies, Baden Daettwil, Switzerland), using a 120 W lamp with a diffuser for illumination (see Fig 1), and a calibration scale positioned in the focal plane. We performed ten trials on a single stamen for each of *Nicotiana glauca*, *Penstemon sp*. and *Aloe arborescens*, and carried out five trials on the stamen from a *Hemerocallis* daylily hybrid. From recorded video sequences, we digitized anther position during the process of electrical attraction using custom tracking software (see Hedrick, 2008). The distance to the model at the initiation of rapid motion of the anther due to electrostatic attraction (as compared to its initially slow displacement toward the model as induced by the experimenter) was recorded, and its average bending speed was estimated as this distance divided by the subsequent time to model contact.

### Particle attraction

We also used the charged hummingbird model to determine if free-floating particles similar in size to pollen grains would exhibit electrostatic attraction. We constructed an artificial stamen using a metal wire (radius: 0.2 mm; length: 2 cm) and dusted the tip with *Lycopodium* spores (~15 micrometers in diameter). Charging of the hummingbird model was performed using the metal-tipped rachis and the Van de Graaff generator described above. Electrical charge was measured by induction using a metal pail connected to the charge sensor; the charged metal tip of the rachis was introduced into and contacted the pail both before and after we touched the model. The difference between these two charge measurements was assumed to indicate the electrical charge transferred to and remaining on the model.

In experimental trials, the artificial stamen was first placed close to the forehead of the hummingbird model. We then gently touched the artificial stamen with a grounded metal pin, which caused the spores to detach and fall from the wire. All trials were recorded using a high-speed video camera (AOS Technologies) operated at 1,000 frames/s. In one set of experiments, the model was charged repeatedly (with a total of 15 events) to values characteristic of free-flying hummingbirds (see below), and the distance between particle and model at which electrostatic attraction became evident was measured. We also digitized the path of five haphazardly chosen particles attracted to the model when it was charged at 490 pC, and also at 840 pC. As a control, we measured trajectories for ten particles in the presence of a grounded and thus uncharged model. Particle velocity and acceleration were calculated as the first and second derivative of positional displacement curves, as smoothed by a quintic spline [8]. Velocity and acceleration differences between the two charged treatments and the control condition were evaluated using a Wilcoxon rank-sum test.

### Wing rubbing

We investigated triboelectric effects produced by first fastening a first grounded and then isolated hummingbird wing (obtained from a salvage specimen of a male *C*. *anna*; Museum of Vertebrate Zoology, UC-Berkeley) onto the tip of a cut rachis of a pigeon feather. The isolated wing was rubbed by hand at ~10 Hz against either the flower petals or the leaves of a tea plant (*Camellia sinensis*) for about four seconds by a grounded observer. We measured the charge of the wing before and after the rubbing via introduction into the aforementioned metal pail as connected to the charge sensor; the difference between these two measurements was taken to indicate the net charge transferred to the wing by friction. We performed ten repetitions of this measurement sequence each for rose petals and for rose leaves.

## Results

### Hummingbird electrostatic charge

**Fig 2:**
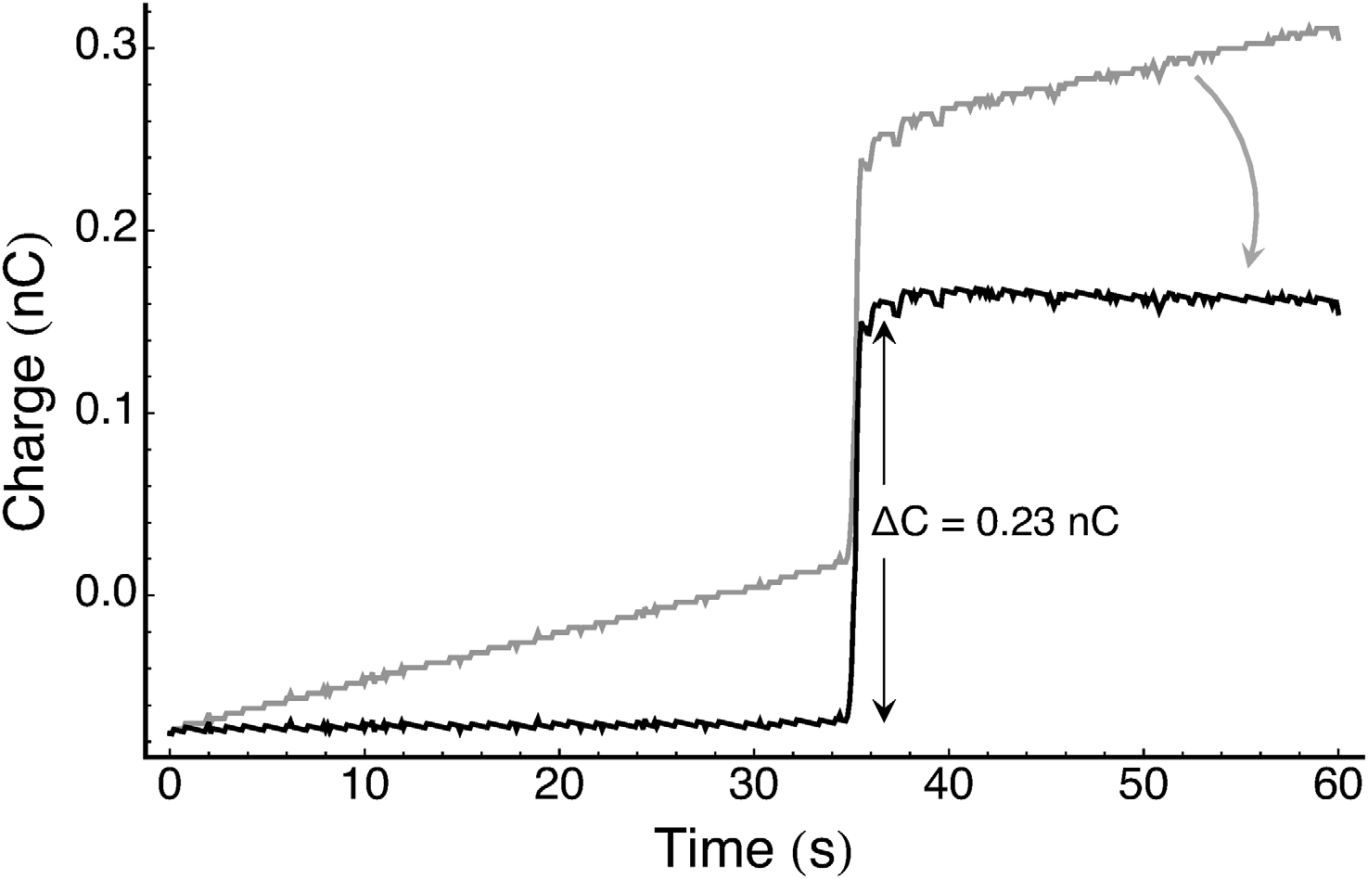
Representative charge measurement on a free-flying wild hummingbird. Sensor traces were corrected for baseline drift; Δ indicates the measured bird charge.

**Fig 3:**
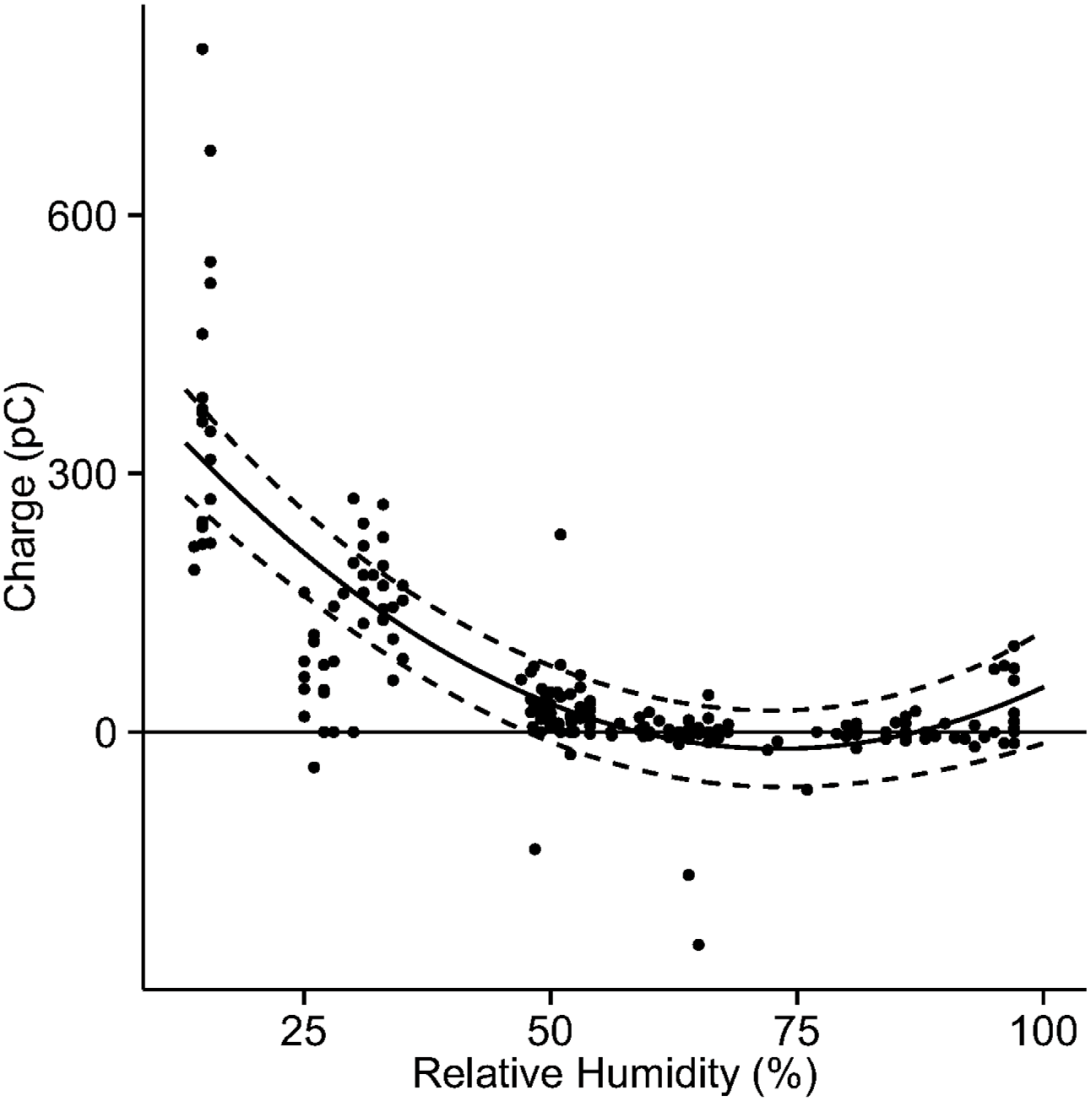
Hummingbird charge as a function of relative humidity; each point represents a single measurement. The solid curve shows the best-fit quadratic curve: *C* = (505 ± 39) + (-14.4 ± 1.5) *H* + (0.098 ± 0.013) *H* ^2^, where *C* is charge in pC and *H* is relative humidity (%); parameter estimates for fixed effects are expressed as means ± 1 standard error. Dashed lines represent the 95% confidence band, taking into account uncertainty in both fixed and random effects.

Free-flying hummingbirds exhibited charges ranging from -250 pC to +800 pC, and averaged 66 ± 129 pC (n = 194 observations; see Fig 2). Air temperature and relative humidity for these same observations averaged 16 ± 2°C and 55 ± 24%, respectively. Net charge decreased significantly with humidity (Fig 3; likelihood ratio test, *F2* = 6.70, *p* = 0.035), but did not vary with air temperature (likelihood ratio test, *F2* = 1.75, *p* = 0.42). The highest charges were recorded during moderately windy days at relative humidities of 14–16%.

### Stamen attraction

**Fig 4:**
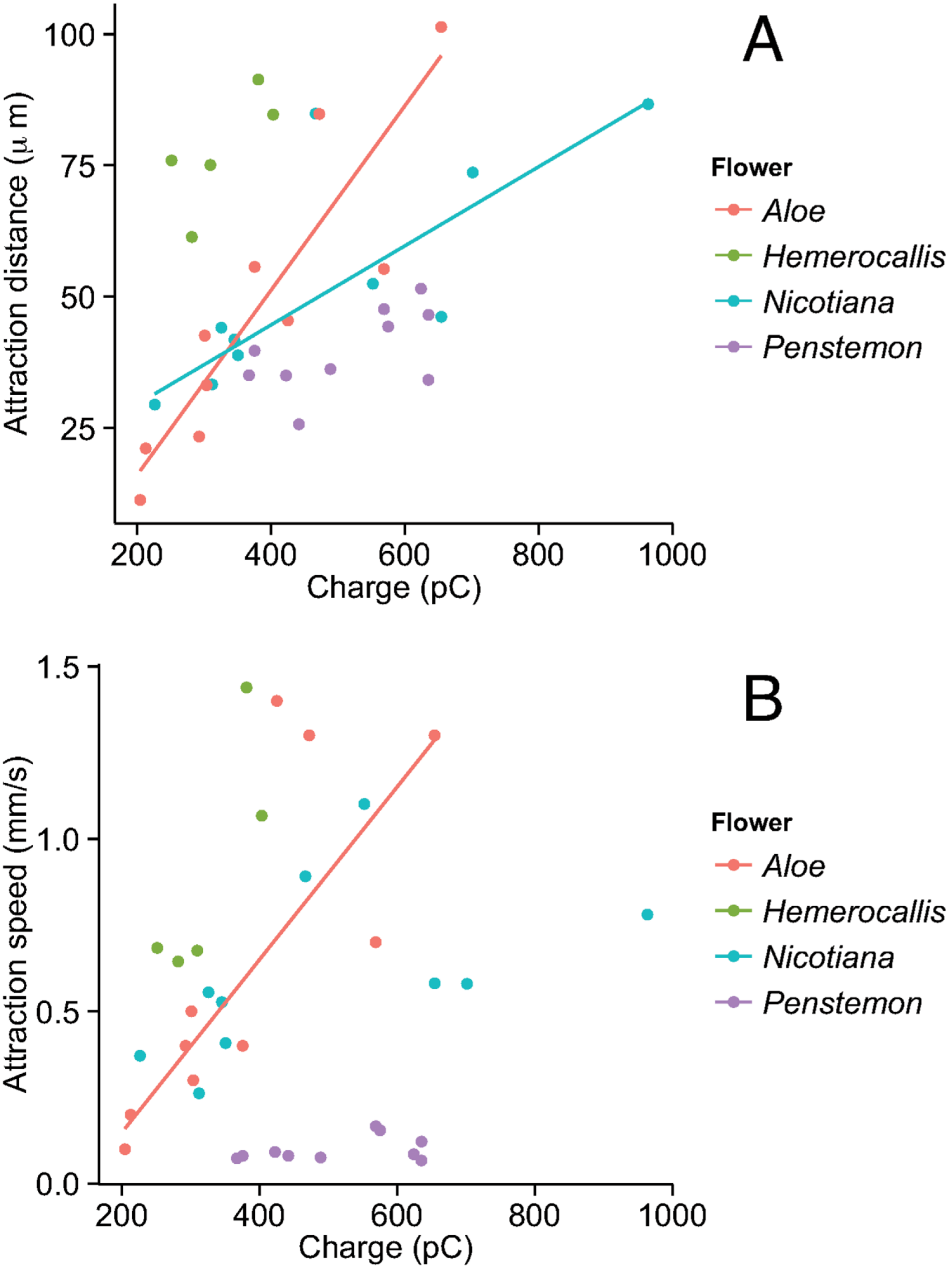
Attraction distance in μm (A) and mean speed in mm/s (B) of floral anthers produced by a metal hummingbird model with varied electrostatic charges (in pC). Linear regressions as follows: *Nicotiana glauca* (red symbols, n = 10; *distance* = 18 + 0.07 × *charge*, *p* < 0.01; *speed* = 0.33 + 0.0006 × *charge*, *p* = 0.13), *Hemerocallis* sp. (green symbols, n = 5; *distance* = 35 + 0.13 × *charge*, *p* = 0.15; *speed* = -0.53 + 0.0044 × *charge*, *p* = 0.09), *Penstemon* sp. (blue symbols, n = 10; *distance* = 18 + 0.04 × *charge*, *p* = 0.08; *speed* = 0.03 + 0.0001 × *charge*, *p* = 0.26), *Aloe arborescens* (purple symbols, n = 10; *distance* = -17 + 0.17 × *charge*, *p* < 0.001; *speed* = -0.32 + 0.0026 × *charge*, *p* < 0.001).

Depending on charge, stamens from all four tested plant species were attracted to the metal hummingbird model at distances between 10–100 μm (Fig 4A, Table 1). Stamens from *Nicotiana*, *Penstemon*, and *Aloe* bent along their filament, whereas stamens from *Hemerocallis* exhibited no obvious bending. The anthers of *Hemerocallis* and *Aloe* also rotated about the connection with their filament to make contact with the hummingbird model. Attraction distance increased significantly with model charge (likelihood ratio test, F_1_ = 38.2, p < 0.001; Fig 4A), but also varied significantly with on floral species (likelihood ratio test for charge × flower interaction, F_3_ = 4.11, p = 0.016). Increased model charge generally increased speeds of attraction (likelihood ratio test: F_1_ = 12.5, p = 0.001; Fig 4B), but this effect also depended on flower species (likelihood ratio test for charge × flower interaction: F_3_ = 5.24, p = 0.006). Model charge had little effect on the attraction speeds of *Penstemon*, values of which were ~90% lower than those of the other species (Fig 4B, Table 1, S1 Video).

**Table 1:** Displacement distances and speeds for electrostatic attraction of different flower anthers to a metal hummingbird model of varying charges. Data indicate mean values ± one standard deviation.

species	charge (pC)	distance (μm)	speed (mm/s)
*Nicotiana* sp. (n = 10)	490 ± 228	53 ± 21	0.61 ± 0.25
*Hemerocallis* sp. (n = 5)	325 ± 65	78 ± 11	0.90 ± 0.35
*Penstemon* sp. (n = 10)	513 ± 107	40 ± 8	0.10 ± 0.04
*Aloe* sp. (n = 10)	381 ± 149	47 ± 28	0.66 ± 0.49

### Particle attraction

**Fig 5:**
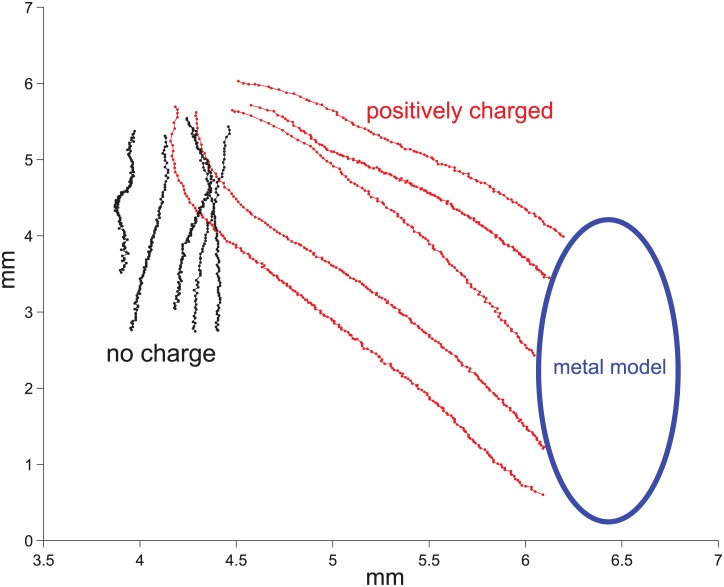
Attraction of *Lycopodium* spores to a metal bird model when positively charged at 450 pC (red trajectories), and spore displacement with no net charge on the model (black trajectories).

We observed electrical attraction of *Lycopodium* spores to the hummingbird model at charges of 573 ± 208 pC (n = 15), with an average separation distance of 1.6 ± 0.5 mm (Fig 5, S2 Video). At model charges of 490 pC and 840 pC, falling *Lycopodium* spores were attracted laterally towards the model at horizontal speeds of 13 ± 4 mm/s (n = 5) and 13 ± 3 mm/s (n = 5), respectively, which were significantly greater than the -6 ± 6 mm/s control speeds (Wilcoxon rank-sum tests: 490 pC, W = 25, p < 0.001; 840 pC, W = 25, p < 0.001). Horizontal speeds between the two charged treatments were not significantly different (W = 12, p = 1), but were more than twice those of the uncharged case (Table 2). When the hummingbird model was grounded, *Lycopodium* spores fell vertically at a speed of -34 ± 13 mm/s (n = 10). This component of speed was similar for the charged and uncharged cases (490 pC vs. control, W = 13, p = 1; 840 pC vs. control, W = 20, p = 0.151). We found no significant differences for either horizontal (W = 7, p = 0.3) or vertical (W = 15, p = 0.69) spore accelerations between the charged model at 490 pC and the uncharged model. By contrast, horizontal accelerations of the spores were significantly greater for the 840 pC case as compared to the uncharged case (W = 25, p = 0.01), but the corresponding vertical acceleration was unchanged (W = 13, p = 1).

**Table 2:** Vertical and horizontal components of speed and acceleration for *Lycopodium* spores drifting near a metal hummingbird model with two different charges, and when uncharged. Data indicate mean values ± one standard deviation.

trial	speed (mm/s)		acceleration (mm/s^2^)	
	horizontal	vertical	horizontal	vertical
490 pC (n = 5)	13 ± 4	-25 ± 6	-82 ± 95	140 ± 6
840 pC (n = 5)	13 ± 3	-32 ± 6	163 ± 52	168 ± 6
uncharged (n = 10)	-6 ± 6	-34 ± 13	-4 ± 52	95 ± 88

### Wing rubbing

Rubbing a single hummingbird wing against either leaves or flower petals increased its static charge from a nominally zero grounded condition to average values of 620 ± 184 pC and 658 ± 184 pC, respectively (n = 10 in both cases).

## Discussion

Wild Anna's hummingbirds harbor electrostatic charges up to 800 pC while foraging, which is a value much higher than the positive charges described for foraging honeybees (see [3]). Net charge carried by hummingbirds generally decreased with increasing relative humidity, but some charge remained even under conditions near 100% relative humidity (Fig 3). Other environmental conditions may facilitate charge accumulation, such as flight in rain. Raindrops falling through the atmosphere can reach charges of hundreds of picocoulombs [9], and transfer of charge via contact with mist or raindrops is thus feasible for flying hummingbirds, which are known fly effectively even during heavy rain (see [10]).

Because animal pollination of flowers is primarily carried out by volant taxa, these findings, together with documented electric charges on free-flying honeybees [3], suggest that electrostatic pollen transfer to animal vectors may be more common than is currently recognized. At short distances, electrostatic forces can easily surpass in magnitude other forces exerted on pollen grains (e.g., those of gravity and aerodynamic drag). For example, a voltage of ~700 V carried by a tethered bee is sufficient to attract floating pollen, and, when the separation distance is <600 μm, to detach pollen grains attached a substrate [4]. Assuming a bee capacitance of 0.1–0.2 pF (see [4]), this voltage corresponds to an electrostatic charge of less than 100 pC. Similarly, honeybee bodies charged to about 180 pC induce rapid displacement of silk threads when translating near a grounded spider web, thereby increasing the chance of physical contact [11]. The presence of charge may thus influence outcomes of various ecological interactions, even including the remote assessment of pollination status of flowers (see [12]). If we assume for Anna's Hummingbirds a capacitance of 1.1 pF (i.e, a spherical capacitor with a 2 cm diameter) with a charge of 490–840 pC, we then obtain an electrical potential of 440–750 V. The *Lycopodium* spores used in aforementioned experiments were attracted at comparably high voltages, albeit at distances up to 16 mm instead of the 0.6 mm demonstrated for pollen attracted to charged bee bodies. In our experiments the particles were attached to an artificial stamen and were mechanically agitated to effect initial detachment, whereas the experiments with charged bees considered pollen grains naturally attached to the floral anther. Also, we measured both positive and negative charges on wild hummingbirds, in agreement with a prior report of the signs of electrical charge on wild bees [3]. Either positive or negative net charge on flying birds, because of the effects of electrical induction, will elicit similar electrostatic attraction on grounded plant filaments and detached pollen grains.

Triboelectric charges resulting from rubbing of a hummingbird wing on leaves and petals may indirectly derive from the high wax content found in plant cuticles (see [13]). Waxes are known for their high polarization capacity, and have even been exploited to manufacture “electrostatic magnets” known as electrets (see [14]). Triboelectrification has been also studied for insects waking over artificial surfaces; houseflies, with a body mass three orders of magnitude lower than that of a hummingbird, can generate up to 50 pC after waking over a polyvinyl chloride surface, but charge to only 10 pC after walking over an acetate surface [15]. Triboelectrification can potentially effect charging of both invertebrates and vertebrates moving on or through vegetation, although no studies are available that directly address this point. Plant waxes and animal oils may particularly influence triboelectric charging. Note that, whereas the manual rubbing used here only roughly mimics any natural interaction between hummingbirds and plant structures, we obtained a charge up to 700 pC, i.e., only 13% lower than the maximal charge measured on wild birds. We accordingly suggest that hummingbirds can become electrically charged through friction with plant structures and air particles (ions, dust, ash and water droplets), and even during self-grooming or via intra-specific contact.

Electrostatically induced bending of floral stamens (Fig 4, Table 1) may facilitate physical contact of the anther onto a hummingbird, and thereby promote adhesion of pollen grains. Pollen detachment and subsequent attachment onto an animal vector is more likely if adhesion between the pollen and the anther is low, and if attachment force to the pollinator is high. Stamen bending also is more likely for flowers with thin and flexible filaments, and for those with mobile anthers. Electrostatic attraction of pollen onto bees has been experimentally observed [4], but it is unknown if the anther displacement observed in our experiments also can be induced by small insects. Vibratory pollen-collection is a common practice of bees to obtain pollen from certain pendent flowers (see [7]), and airflow induced by a hovering hummingbird (together with beak motions) may similarly vibrate floral structures to release pollen. For hummingbirds, the downwash produced during hovering and the fore and aft movements of the beak during feeding may also vibrate flower structures and mechanically eject pollen. Once free of the anther, electrostatic forces can also facilitate the recapture of neutral floating grains by a charged animal. In the presence of an external electric field, neutral grains develop a dipole, which attracts negative ions from the surrounding air to its positive pole [16]. Through this process, grains may quickly acquire a negative charge and experience a net force. As shown in our experiments with *Lycopodium* spores (Table 2), even neutrally charged particles can be attracted by a nearby positively charged object. Neutral pollen grains falling from the anther could thus be attracted at distances of several millimeters by a net charge as low as ~400 pC. The relative importance of electrostatic and vibratory mechanisms of pollen detachment may also vary according to typical levels of environmental humidity (e.g., arid versus humid habitats), and potentially by different types of animal pollinators.

A typical Reynolds number for *Lycopodium* spores (15 μm in diameter) moving in air at 13 mm/s is about 0.01, a flow regime for which viscosity is dominant and for which a high electrical potential is required to overcome Stokesian drag. For example an electrical potential of 300 × 10^5^ V (three orders of magnitude higher than that used in our experiments) is required to move dust particles at 1 m/s (see [16]). In our experiments, *Lycopodium* particles started with a initial non-zero speed because they were vibrated off from an artificial anther, and thus drag acted immediately to decelerate the particle. The irregular shape of a hummingbird (or model) must also influence particle kinematics because the spatial electrical field depends on the geometry of the charged object. Because of these effects, the speeds and accelerations attraction of these particles must be viewed cautiously, although the charges used were certainly comparable to those measured on wild hummingbirds.

In conclusion, our results indicate that wild hummingbirds carry electrostatic charges high enough to displace floral stamens over short distances, and also to attract free-floating pollen grains. Charges on free-flying animals more generally are not well characterized, but may influence the effectiveness of pollination behavior. We accordingly suggest that broader surveys relating electrostatic charge to body size and to environmental variables, particularly relative humidity, be conducted under natural circumstances.

## Supporting Information

S1 Video Electrical attraction of floral structures. Electrical attraction of floral anthers produced by a metal hummingbird model charged to 552 pC, 403 pC, 425 pC and 635 pC (*Nicotiana glauca*, *Hemerocallis* sp., *Aloe arborescens*, and *Penstemon* sp., respectively).
(MP4)

S2 Video Electrical attraction of floating particles. Electrical attraction of *Lycopodium* spores to a non-charged and charged (490 and 840 pC) metal bird model.
(MP4)
